# A Case of Para-Bombay Phenotype Caused by Homozygous Mutation of the FUT1 Gene

**DOI:** 10.4274/tjh.2017.0220

**Published:** 2017-12-01

**Authors:** Jung-Kuang Yu, Yi-Hong Liu, Tze-Kiong Er

**Affiliations:** 1 Asia University Hospital, Department of Orthopedics, Taichung, Taiwan; 2 Asia University Hospital, Division of Laboratory Medicine, Taichung, Taiwan; 3 Asia University Faculty of Medicine, Department of Health and Nutrition Biotechnology, Taichung, Taiwan

**Keywords:** Para-Bombay, Phenotype, FUT1 gene, Blood transfusion

## To The Editor,

**Figure 1:**
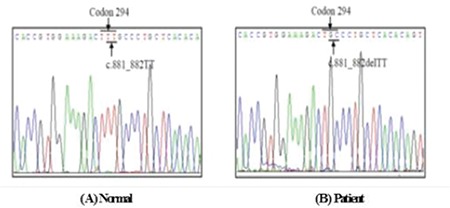
Sequencing results confirm the wild-type (A) and the presence of the FUT1 homozygous mutation c. 881_882delTT (p. Phe294Cysfs*40) (B).

A 79-year-old female patient presented at the hospital with osteoarthritis. Examination of the patient revealed hemoglobin level of 10.8 g/dL, RBC count of 3.45x10^6^/µL, WBC count of 10.1x103/µL, and platelet count of 122x10^3^/µL. Plasma levels of blood urea nitrogen, creatinine, sodium, potassium, and alanine aminotransferase were all within the normal ranges, while aspartate aminotransferase was slightly higher than normal. A blood sample obtained from the patient was submitted to our division for blood typing and cross-matching, with a request to receive 2 units of packed red blood cells. ABO typing was performed using standard serological techniques after an immediate spin. Testing the patient’s red blood cells revealed no detectable ABO antigens upon forward/cell grouping (group O blood type). On the other hand, reverse/serum grouping showed the presence of A antibodies in the serum (group B blood type). To resolve the discrepancy between cell and serum grouping we performed an agglutination examination of anti-H serum; the red blood cells from the sample did not exhibit an agglutination reaction. Additionally, secretor status was determined in order to assess the presence of soluble blood group substances. Our results showed the presence of B and H antigens in the saliva. Based on these results, the patient in the present case was diagnosed as having a para-Bombay B phenotype (Table 1, Figure 1).

Genotyping of the ABO and FUT1 genes was also performed. Direct DNA sequencing of the patient’s ABO gene indicated the B/O1 genotype. To examine potential mutations in the FUT1 gene, we amplified and sequenced the full coding region of the gene. FUT1 gene sequence analysis revealed that the patient harbored the homozygous mutation c.881_882delTT (p.Phe294Cysfs*40). A heterozygous mutation in FUT1 (880delTT) has been previously reported as the cause of the para-Bombay phenotype [1,2]. However, the homozygous mutation c.881_882delTT (p.Phe294Cysfs*40) only rarely causes the para-Bombay phenotype. Previously, a study indicated that homozygous mutations are a cause of the para-Bombay phenotype [3,4].

In patients with the para-Bombay blood group, ABH antigens are present in saliva but not expressed in red blood cells. The para-Bombay phenotype results either from an inactive FUT1 gene present together with a normal FUT2 gene or from a mutated FUT1 gene present with or without an active FUT2 gene [1]. H deficiency is slightly more common in Taiwan, affecting 1 of 8000 people [2]. More than 56 silencing or weakening FUT1 mutations have been reported in the dbRBC database (https://www.ncbi.nlm.nih.gov/projects/gv/mhc/xslcgi.cgi?cmd=bgmut/systems_info&system=hh).

In conclusion, identification of this phenotype is very important because this particular patient subgroup may be clinically mislabeled as group O. If patients with anti-H in their circulation receive transfusions of blood with the H antigen, it may cause a transfusion reaction such as an acute hemolytic reaction. Here we have reported a rare case of the para-Bombay phenotype caused by the homozygous mutation c.881_882delTT (p.Phe294Cysfs*40).

## Figures and Tables

**Table 1:**
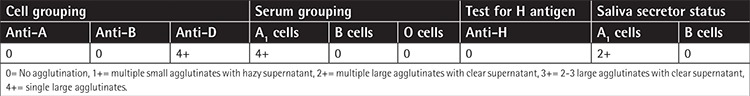
Serologic and saliva test results of the patient: ABO group discrepancy.
